# Use of a mobile plant identification application and the out‐of‐school learning method in biodiversity education

**DOI:** 10.1002/ece3.10957

**Published:** 2024-04-17

**Authors:** Ozan Coşkunserçe

**Affiliations:** ^1^ Faculty of Education, Computer Education and Instructional Technologies Department Nevşehir Hacı Bektaş Veli University Nevşehir Turkey

**Keywords:** biodiversity education, mobile application, out‐of‐school learning, plant identification

## Abstract

Today, many students are no longer able to identify plants and researchers use the term “plant blindness” to describe students' ignorance of plant species. Knowledge of plant species is among the factors that best support an interest in and understanding of environmental issues, biodiversity, and a sustainable lifestyle. With the help of mobile technologies, it is thought that the knowledge level of students about herb and tree varieties can be increased outside of class hours and in outdoor education. The aim of this study was to determine the effect of the use of the PlantNet mobile application and the out‐of‐school learning method on the knowledge levels of 5th‐grade students about the plant species in their environment and their behaviors demonstrating an understanding of biodiversity. For this purpose, at the beginning of the study, a plant species questionnaire and a biodiversity behavior questionnaire were applied to the students. Afterward, the students were asked to examine the plant species around them using the PlantNet mobile application. At the end of the activities, the data collection tools applied at the beginning of the study were applied again. It was determined that the students who participated in the activities displayed more biodiversity‐related behaviors than before they participated in the activities, and that the students were able to write down more herb and tree species at the end of the activities. In addition, as a result of the activities, a positive and high‐level relationship was found between the students' biodiversity‐related behaviors and the total number of plant species they knew.

## Graphical abstract

With the help of mobile technologies, it is thought that the knowledge level of students about plant and tree varieties can be increased outside of class hours and in outdoor education. The aim of this study was to determine the effect of the use of the PlantNet mobile application on the knowledge levels of 5th‐grade secondary school students about the plant species in their environment and their behaviors, demonstrating an understanding of biodiversity.

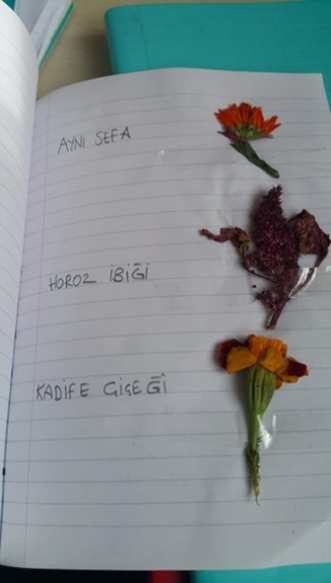

## 1 INTRODUCTION

Biodiversity is a concept coined in the mid‐1980s by naturalists who were concerned about the increasing destruction of wildlife, particularly rainforests (Lévêque & Mounolou, 2004). The simplest definition of the concept of biodiversity is the “diversity of organisms in relationship with each other” (Gaston & Spicer, 2013). Nowadays, environmental problems such as environmental pollution, the extinction of plant and animal species, invasive species, and global warming are increasing concerns. In order to attract more attention to biodiversity issues, May 22 was declared the day of biodiversity by the United Nations (UN, 2022). According to the 2019 “Global Assessment of Biodiversity and Ecosystem Services” report, due to the widespread human impact on various ecosystems, the global rate of species extinction is already at least tens to hundreds of times higher than the average rate over the last 10 million years, is rising steadily, and on average 25% of species are threatened (IPBES, 2019). This pressure on living species is particularly felt on plant species. The rapid increase in the human population in recent decades has triggered a demand for more crops and wood, leading to a reduction in green areas and unprecedented levels of decline in plant species (Fujiwara & Matoh, 2009).

Today, biodiversity and issues related to biodiversity are increasingly on the agenda, and more attention is being paid to biodiversity, especially in the field of science education. The increasing human population and related consumption needs on Earth increase the pressure on biodiversity, and the rapid extinction of species is becoming an important problem. In countries where children and adults have little contact with the natural environment, the public is uninterested in understanding local environmental problems and ecosystems (Evans et al., 2007). Conversely, people who interact with animals and plants at a young age and have spent a lot of time in nature are more aware of environmental problems in adulthood (Erökten, 2006). However, many students can no longer identify plant species (Bebbington, 2005; Braun et al., 2010; Kaasinen, 2019; Lindemann‐Matthies & Bose, 2008; Lückmann & Menzel, 2014; Palmberg, 2012), and researchers use the term “plant blindness” to describe this ignorance about plants (Lindemann‐Matthies & Bose, 2008; Wandersee & Schussler, 2001). Wandersee and Schussler (1999) define plant blindness as “the inability to see or notice the plants in one's own environment, the inability to recognize the importance of plants in the environment and human affairs, the inability to appreciate the aesthetic and unique biological features of plants, and the tendency to rank plants as inferior to animals” (p. 84). Without the ability to identify plant species and without understanding the meaning of plants, it is difficult to protect them or understand how ecosystems and nature work (Kaasinen, 2019). The vast majority of the world's population currently resides in urban areas, and the degree of urbanization has negatively impacted the frequency of direct experiences with nature (Soga et al., 2018). Students' inability to notice plant species in their immediate environment can be cited as a reason for plant blindness, but even in big cities, there are many plant species surrounding them. Among the reasons for this situation is that students, who now spend significant amounts of time in the digital world, lead lives away from nature (Louv, 2006; Özden, 2008) and thus have a lack of interest in plants (Lückmann & Menzel, 2014). The fact that students do not recognize plant species or even realize that they are there is mostly due to the fact that very little time at school is spent in direct observation of plants (Amprazis et al., 2019; Barker, 2002; Lindemann‐Matthies, 2006).

Therefore, educating students about different plants is very important to inform them about biodiversity and to prevent plant blindness. Moreover, identifying plant species and having a basic understanding of their life cycle has been identified as a crucial aspect of biodiversity education (Lindemann‐Matthies, 2002; Palmberg et al., 2019). Many researchers have organized various training programs in schools to introduce plants and increase interest in them (Cil, 2016; Frisch et al., 2010; Krosnick et al., 2018; Lindemann‐Matthies, 2002, 2006; Pany & Heidinger, 2014; Strgar, 2007). One of the main challenges in biodiversity education is thought to be reconnecting humans with nature. For this reason, researchers have argued that biodiversity education should focus on activities that increase students' contact with nature (Katili et al., 2021; Navarro‐Perez & Tidball, 2012). Observing plants and animals in the environment is the first step for students to learn about biodiversity (Wolff & Skarstein, 2020) and in species identification studies with students, outdoor teaching and learning methods were found to be more effective than their indoor counterparts (Palmberg et al., 2019). Field studies are very important for students to gain knowledge through personal observation and practical experience (Palmberg et al., 2015). Similarly, it has been observed that students living in rural environments and those spending more time in nature have better knowledge about plant species (Kaasinen, 2019; Lückmann & Menzel, 2014; Patrick & Tunnicliffe, 2011). Studies for plant species identification have been carried out in different settings such as a botanical garden (Lückmann & Menzel, 2014; Sanders, 2007), in field study courses (Bebbington, 2005; Weilhoefer & Schmits, 2022), as outdoor education in a meadow (Fančovičová & Prokop, 2011), during daily walks to school (Lindemann‐Matthies, 2002) and at school (Kaasinen, 2019). In classic plant identification studies, plant species can be presented to students in the form of living samples (Fančovičová & Prokop, 2011; Sanders, 2007), photographs (Kaasinen, 2019; Palmberg et al., 2015) and illustrations (Bebbington, 2005; Lückmann & Menzel, 2014).

Lack of time and resources is a major obstacle to integrating biodiversity studies into the core curricula of schools (Gayford, 2000). In addition, many of the teachers have not received strong botanical training and need to be supported in this regard (Stroud et al., 2022). It has been suggested that informal learning environments such as botanical gardens and students' home environments can contribute to students' learning about plant species (Sanders, 2007). However, many cities do not have botanical gardens and students living in big cities are less likely to have or live near gardens. In this regard, it is thought that information and communication technologies (ICT) can be used as an effective tool to provide biodiversity education in out‐of‐school and informal learning environments, to offer the opportunity to work outside of class hours and to keep students active in the learning environment. The use of informal learning environments for formal (school‐based) learning is defined as “out‐of‐school learning” (Salmi, 1993). Activities carried out by students in out‐of‐school environments and in nature positively affect their knowledge about plant species (Evans et al., 2006; Fančovičová & Prokop, 2011; Palmberg et al., 2015; Randler, 2008). Biodiversity education on plant species can be transferred to informal learning environments, and out‐of‐school learning activities can be instigated using mobile technologies, which almost every student can access through their parents.

The aim of this study was to determine the effect of the activities carried out with the PlantNet mobile application on 5th‐grade students' behaviors, demonstrating an understanding of biodiversity and their knowledge about plant and tree species in their environment. Within the framework of this aim, answers were sought to the following research questions:

1. How do the activities carried out with the use of the PlantNet mobile application affect 5th‐grade students' biodiversity conservation and interest behaviors?
2. How do the activities carried out with the use of the PlantNet mobile application affect the knowledge of 5th‐grade students about the common names of herb and tree species in their environment?

### 1.1 New techniques in plant identification

New techniques and algorithms have aided in the identification of plant taxonomy as a result of advancements in computer technology, specifically in image processing and pattern recognition (Kho et al., 2017). The combined use of online and easily accessible big data, together with the development of technologies such as machine learning, could provide an unprecedented opportunity to improve trait‐based identification of plants (Almeida et al., 2020). With these technological advancements, computer scientists and botanists have collaborated to create automated plant identification systems. Traditional methods for plant identification are laborious, time‐consuming, and prone to misidentification, making it a difficult task for botanists, nature enthusiasts, and individuals without specialized training (Bojamma & Shastry, 2021). Automated plant identification systems allow nonspecialists to identify plants, making the process much simpler and quicker.

Mobile applications for automated plant identification are widely used today. Many mobile applications have been developed that can be used to identify plant species using photos, and Bilyk et al. (2020) list the most widely used of these applications as follows: Google Lens, PlantNet, LeafSnap, Seek by iNaturalist, Flora Incognita, PlantSnap, and Picture This. According to the results of this study, Flora Incognita and PlantNet have the most useful and informative interfaces among the plant identification applications, while Google Lens is the most successful application in terms of 92.6% plant identification accuracy. In the comparison of plant recognition applications in terms of the identification of 55 tree species found in the United States, PictureThis application was determined to be the most successful application with 67.8% correct result (Schmidt et al., 2022).

Among these applications, iNaturalist is very popular among researchers and has been used in a large number of studies. Unger et al. (2021) observed that university students who used the iNaturalist mobile application to identify organisms increased their ability to identify local biodiversity and their engagement in biological sciences. In another study using the iNaturalist application, undergraduate science students reported that the education they received in an out‐of‐school learning environment was successful and that they had learned new information about species identification (Gass et al., 2021). In another study, the iNaturalist mobile application was used by secondary school students in collaborative learning activities, and they were reported to have positive opinions about the application (Echeverria et al., 2021). Di Cecco et al. (2021) examined user behavior on the iNaturalist platform and found that users specialized in certain areas, with the most common areas of study being plants and insects. In Forti (2023) study, it was shown that 93.3% of university students who utilized the iNaturalist application indicated a significant enhancement in their quantitative understanding of biodiversity. Menezes Neto et al. (2021) developed the NEIDE application, which used artificial intelligence to identify plants from plant photos in the database.

Although the PlantNet mobile application lags behind other mobile applications with a plant identification success rate of 55% (Bilyk et al., 2020) and more than 60% (Xing et al., 2021), its genus suggestion success rate of 88.41% and species suggestion success rate of 70.68% are quite successful (Schmidt et al., 2022) and stand out in comparison to other features. In various comparisons of mobile plant identification applications, PlantNet was determined to be the most successful because it is free, supports 45 languages, including Turkish, is simple to use, and offers highly accurate and rapid identification (Airhart, 2023; Baker, 2023). The ability of the PlantNet mobile application to use various plant organs (leaf, flower, fruit, etc.) for plant identification is cited as a competitive advantage over other applications (Affouard et al., 2017). PlantNet is recommended for use for educational purposes as it increases students' motivation to investigate nature (Bilyk et al., 2020). Activities utilizing the PlantNet mobile application provide more permanent information about plant species than computer‐based digital herbarium activities (Iskrenovic‐Momcilovic, 2020). As a result of plant identification activities conducted with PlantNet, it was observed that students' plant identification skills improved significantly (Santri et al., 2021), and a significant improvement was observed in students' awareness and levels of concern about environmental problems (Álvarez‐Herrero, 2023). Secondary school students used PlantNet, traditional image‐based printed identification keys, and unstructured searches on the Internet to identify plants and stated that PlantNet was the least difficult of these methods (Lang & Šorgo, 2022). Similarly, high school students identified the PlantNet application as a successful tool for identifying plant species, and it was observed that they had learned more as a result of this activity (Muchsin et al., 2021). Meanwhile, it was emphasized that the taxonomy of some plants does not correspond to their actual names in PlantNet identifications, and therefore users should be more cautious when identifying plant species with PlantNet (Santri et al., 2021). Although the students chose PlantNet as the method with which they encountered the fewest difficulties in plant identification, they indicated that books would be their future method of choice for species identification (Lang & Šorgo, 2022).

## 2 METHOD

### 2.1 Research design

The study employed a one‐group pretest‐posttest research design, which is a quantitative research method. According to Büyüköztürk et al. (2008), “studying with a single subject allows the independent variable to be measured better, the data to be collected and interpreted more quickly, and if necessary, the independent variable and, if any, its levels can be revised to include these effects in the research” (p. 207). “Although a change from pretest to posttest can be due to the intervention, there are many possible extraneous factors to be considered” (McMillan, 1996, p. 217). For this reason, the data on the source of the change between the pretest and the posttest were collected within the scope of the second research question, and it was investigated whether the change occurred due to the intervention. Moreover, the test‐retest method was utilized to improve the validity of the research findings. The test‐retest method involves administering the same test twice to the same group after an equal time interval and calculating a reliability coefficient to indicate the relationship between the two scores obtained (Büyüköztürk et al., 2008). Good test‐retest reliability is indicative of a test's internal validity and ensures that the measurements obtained in a single sitting are both representative and stable over time. For this reason, 4 months after the posttest was administered, the data collection tools were administered again as a retest. The Pearson correlation coefficient was computed to assess the linear relationship between the posttest and retest QBB scores.

The one‐group pretest‐posttest design has been criticized, especially for internal validity issues. The primary rationale for selecting this research model is the impracticality and lack of significance of establishing a control group within the study's framework. The curriculum for fifth‐grade science and other classes lacks any activities aimed at enhancing students' biodiversity‐related behaviors and understanding of plant species. Consequently, no change in the behavior and understanding of students regarding biodiversity and plant species in the control group is expected to occur between the pretest and posttest. Furthermore, the absence of a control group might be attributed to the fact that there are no alternative methods available, apart from mobile applications, to accurately identify plants within this specific age range. Due to the difficulty and extensive training required, other plant identification methods are not suitable for students in this age group.

The one‐group pretest‐posttest paradigm acknowledges that certain factors pose a risk to internal validity (Price et al., 2015) and that the disparity between pretest and posttest scores may be attributed to factors beyond the intervention itself. The occurrence of additional circumstances that lead to a difference between the pretest and posttest, namely from the pretest to the posttest, are referred to as history effects. No activities unrelated to the study on biodiversity and plant species were undertaken in science and other courses at the students' school during the period between the pretest and posttest. To identify the origin of the shift in students, data was gathered using a distinct questionnaire. Additionally, the students were inquired about the source of their knowledge regarding the names of herb and tree species subsequent to the exercises. Most students cited the PlantNet program and their teachers as the primary sources from which they acquired new knowledge of plant names. Another factor is that participants may have experienced alterations in their composition between the pre‐test and post‐test due to the process of maturation and the acquisition of knowledge. The duration between the pre‐test and post‐test is insufficient for students to have sufficiently progressed in order to perform better on the post‐test. Furthermore, it should be noted that the preceding paragraph elucidated the notion that actions serve as the primary catalyst for the acquisition of knowledge.

### 2.2 Participants and procedure

The study group consisted of 44 5th‐grade students from a school in the city center of Nevşehir in Turkey who participated in regular plant identification activities using the mobile application. In determining the sample, the typical case sampling method was used from among the nonprobability (purposive) sampling methods.

**FIGURE 1:**
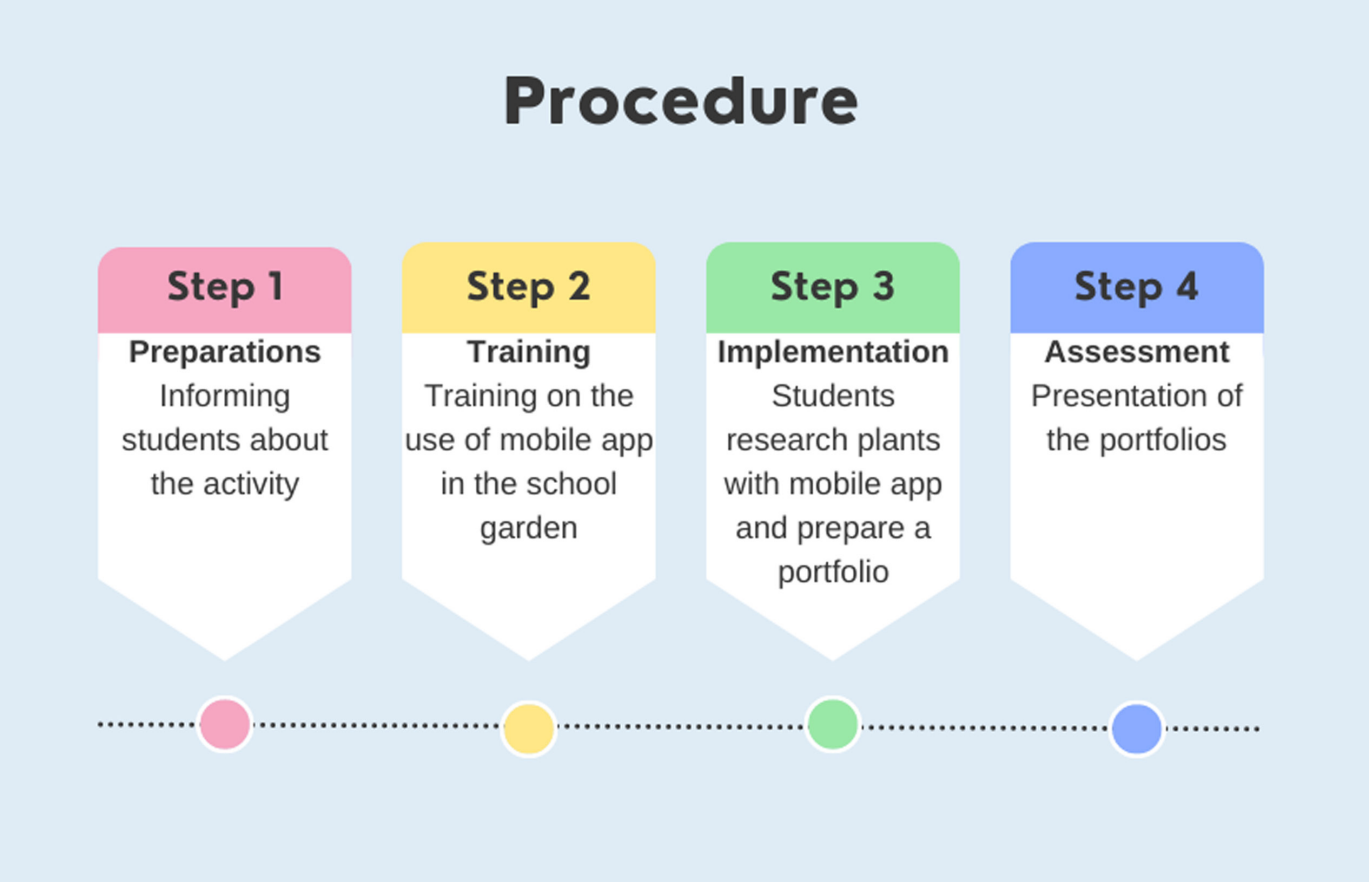
Biodiversity education procedure with out‐of‐school learning methods and a mobile app.

Within the scope of the study, the topic of “biodiversity” in the 5th‐grade science curriculum was supplemented with out‐of‐school learning activities using the PlantNet mobile application. The aim was for the students to achieve the “questions the importance of biodiversity for natural life” learning outcome in the curriculum. The activities were conducted over duration of six class hours. Nevertheless, the entirety of these six class hours was not solely dedicated to the activities, since the usual teaching schedule was still maintained. The out‐of‐school learning method was used in the planning and execution of the activities. The implementation of out‐of‐school learning activities took place in three stages: pre‐activity preparations, implementation of the activity, and post‐activity evaluation (Şen, 2019). The process of the activities carried out within the scope of the study is presented in Figure 1.

In the pre‐activity preparation phase, the science teacher and the researcher worked together to prepare a time and flow plan for the activities. Before the implementation in the school garden, the teacher gave preliminary information to the students in the classroom and explained the rules of the activity. This phase takes up the first hour of the six class hours. Then the activity was carried out. In the second phase of the procedure, students and the teacher went out to the school garden during the class hour. This task was carried out during the second out of six class hours. Using the PlantNet mobile application, numerous plants in the school's garden were photographed and identified. Additionally, students were encouraged to try out the plant identification application. During plant identification, it was emphasized that a match rate above 50% was a more reliable result and that if the match rate fell below 50%, the identification should not be accepted or the teacher should be consulted. The activities carried out in the school garden lasted two class hours and approximately 80 min. After the activity in the school garden, the students were asked to collect samples from herbs and trees in their surroundings and to stick these samples in their notebooks. Students were also informed about how to cut these samples off in order not to harm the development of plants while collecting them. The students identified the species of the specimens they collected by checking them with the PlantNet application, and they wrote the species' names next to the specimens in their notebooks. Students had 3 weeks to complete their research and portfolio. In the third and fourth lesson hours, students who encountered difficulties with plant identification were provided with feedback.

The PlantNet mobile application was used for the students to identify plants. PlantNet was chosen over many other mobile plant identification applications because it is free, supports the Turkish language, is simple and quick to use, and provides plant names, which is the information required for the study, with a user interface suitable for the age group of the participants. PlantNet is an application that allows identifying plants by simply photographing them with a smartphone. In order to identify plants, it is necessary to take a close‐up photo of the plant with clear features and wait for the application to check the photo.

**FIGURE 2:**
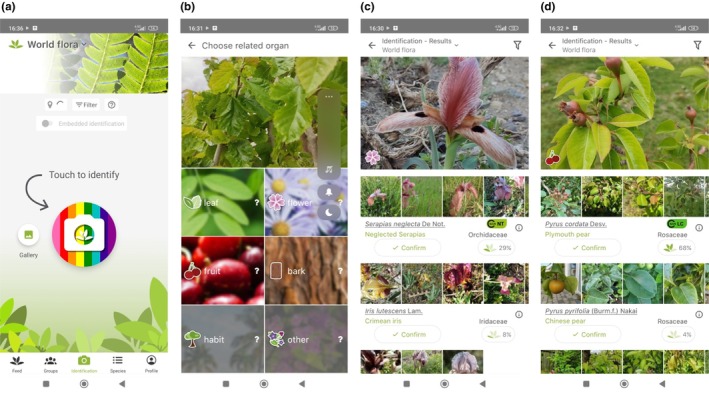
Screens used for plant identification in the PlantNet mobile application.

Figure 2a shows the main screen of the PlantNet application. Here, you can take a photo of the plant to be identified by clicking on the “Touch to identify” icon, or you can identify a plant with a photo by clicking on the “Gallery” icon. In the second stage screen shown in Figure 2b, the type of plant photo must be selected from the leaf, flower, fruit, bark, habit, or other options. If the image of the photographed plant is stored in the application's database, information about the plant is transmitted to the user. Figure 2c shows the plant identification screen of a rare orchid species. Although the plant is identified as “Neglected Serapias” (*Serapias neglecta De Not*.), the match rate is as low as 29%. Figure 2d shows the plant identification screen of a common fruit tree species. The tree is identified as “Plymouth Pear” (*Pyrus cordata*), and the match rate is as high as 68%. As a result, it can be seen that well‐known plant species have a higher matching value. PlantNet mobile applications can be used free of charge on mobile devices with Android and iOS operating systems. PlantNet is also a major citizen science project. All photographed plants are collected and analyzed by scientists around the world to better understand and better protect the development of plant biodiversity. In the PlantNet application, more than 37 thousand plant species can be identified (PlantNet, n.d.). However, the project aims to increase this number to a much higher level in the future. Amateur users' submissions are also reviewed by the community, and those found suitable are published in the species photo gallery.

PlantNet provides additional features in addition to plant identification. For instance, PlantNet provides instructions on how to take an app‐appropriate photograph of a plant, as well as examples of what they do and do not want. Additionally, users can share plant identifications with other users. These characteristics were outlined in the training provided to students at the beginning of the plant identification activities.

**FIGURE 3:**
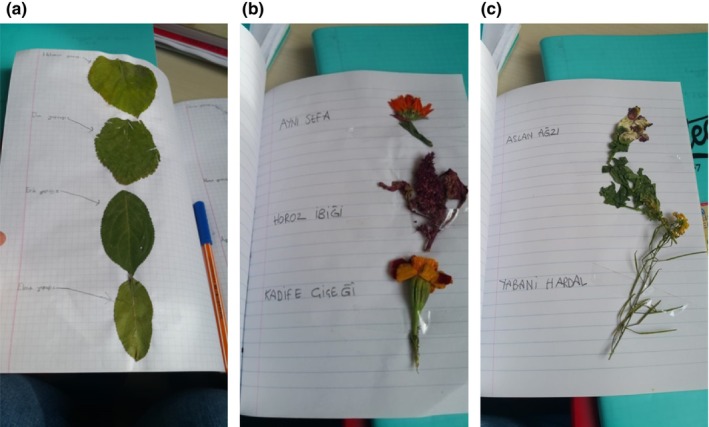
Examples from student portfolios.

Finally, the last stage of the out‐of‐school learning activities was the post‐activity assessment. The importance of the assessment phase for out‐of‐school learning activities has been emphasized, and it has been stated that if the assessment is not carried out, the activity does not go beyond being a simple excursion (Şen, 2019). This phase was conducted during the fifth and sixth class hours. At this point, students brought their prepared notebooks on herb and tree species to school, where their teacher evaluated them with notes. The five students with successful notebooks presented their findings and introduced their classmates to the plants they had identified. In addition, photos and videos of some of the pages prepared by the students were taken and shared on a blog (https://biyolojikcesitlilik.blogspot.com/) and the students were asked to visit the blog. Figure 3 shows examples of the portfolios prepared by the students.

As seen in Figure 3, the species name of the plant identified by scanning with PlantNet was written in the student notebooks next to the samples in a manner that did not harm the plant's development. The students who prepared the portfolios are in the 5th grade; thus, the contents of the portfolios were tailored to be uncomplicated and suitable for the students' cognitive abilities. In the students' notebooks, they wrote common names instead of Latin names. The 5th‐grade science curriculum (Ministry of National Education, 2018) recommends not using systematic names in the classification of living things. Thus, it was ensured that new information was compatible with students' existing knowledge of plants. In addition, the posttest and retests were administered during this phase.

### 2.3 Data collection and analysis

The data for the study were gathered from the Questionnaire on Plant Species (QPS) and the Questionnaire on Biodiversity Behavior (QBB). The QBB used in the study is also a data collection tool developed by Ateş (2010). The questionnaire aims to measure secondary school students' behaviors related to individual and social activities regarding biodiversity. Although the questionnaire items assess a wide range of biodiversity‐related behaviors, these can be broadly categorized as biodiversity conservation and interest. The questionnaire consists of 15 items. The answers to the items are in five‐point Likert type and consists of the following points: (1) never; (2) rarely; (3) sometimes; (4) often; (5) always. The mean score achieved by each student is determined by summing the scores ranging from one to five for each of the 15 items and dividing the result by the total number of questions. The mean score of the participant group with respect to the questionnaire was calculated by averaging the scores of the students. The pretest of the questionnaire was conducted 6 months before the activities. The posttest of the questionnaire was conducted after the activities had been implemented with the mobile application. The QPS and QBB were administered as a retest 4 months after the activities were concluded in order to increase the study's reliability and to determine the level of students' knowledge and behavior regarding biodiversity. The reliability coefficient (Cronbach's alpha) of the QBB was examined with SPSS 22 and found to be 0.72 which is considered a sufficient value.

In the QPS, students were asked the names of the herb and tree species they know with two open‐ended questions. Students were asked to write the common names of plants. Botanically, a tree can be considered a plant, but trees are generally much larger in size than plants, and trees have a thicker and sturdier stem, known as the trunk. Unlike trees, herbs lack a woody stem. Since among the plant species in their environment, students interact with trees the most, data were collected on the tree species known to the students. The form was applied as a pretest and a posttest. In the post‐test conducted after the activities, the participants were also asked from which source they learned the names of herb and tree species with two separate open‐ended questions. As a result, there were two open‐ended questions in the pretest and four in the posttest.

The SPSS 22 software was used to analyze the data. As a result of the skewness and kurtosis analysis and the Shapiro‐Wilk test, it was found that the data obtained from QBB showed a normal distribution, and it was decided to use parametric statistical analysis methods. In addition, Levene's *F* test showed that the variances were equal.

After determining that the data were normally distributed, the pretest and posttest scores of the QBB were analyzed with the paired samples *t*‐test (Data S1). Internal consistency was determined by analyzing the correlation between posttest and retest scores. In addition, the posttest scores of the groups formed according to the effect of the variables in the PIFB on biodiversity were analyzed by the independent sample *t*‐test. Since the number of students who participated in nature camps was very low and the nature club at the school was not functional, the groups formed with these variables were not analyzed. The pretest, posttest, and retest data collected with the QPS were also analyzed within the scope of the study. Following the calculation of the plant names provided by the participants in response to each question, the mean value acquired by the group was computed through the process of averaging these values (Data S1). In addition, in order to analyze the relationship between students' biodiversity conservation and interest behaviors and the total number of plant species they knew after the activities, the correlation values between the mean of QBB posttest scores and the mean of QPS posttest scores of the participants were examined.

## 3 RESULTS

### 3.1 Students' scores from the QBB

This section presents the results related to the first research question and the pretest, posttest, and retest data obtained from the QBB were examined. The scores for the QBB pretest, posttest, and retest can range from one to five. First, a paired samples *t*‐test was applied to test whether there was a significant difference between the pretest and posttest scores.

When Table 1 is examined, it is seen that there was a significant difference between the pretest and posttest scores of the participants of the study for the QBB, *t*(43) = −5.83, *p* = .000, *d* = 1.19. While the scores of the participants for the QBB were *X* = 3.43 before the activities with the mobile application, they increased to *X* = 3.99 after the lessons. The effect size is strong (*d* > 0.8) (Cohen, 1988).

**TABLE 1:** Paired samples t‐test results of QBB pretest and posttest scores.

	*N*	Mean	SD	*t*	*p*	*d*
Pretest	44	3.43	0.47	−5.88	.000	1.19
Posttest	44	3.99	0.46			

The mean retest score was *X* = 4.02 (SD = 0.45). QBB posttest and retest mean scores were found to be strongly positively correlated, *r*(42) = .94, *p* = .000.

### 3.2 Students' knowledge on herb and tree species

This section presents the results related to the second research question and the pretest, posttest, and retest data obtained from the QPS were examined.

Examining Table 2 reveals that the average number of herb species that students could correctly write the name of before the mobile application activities was *X* = 3.5 and increased to *X* = 11.4 after the activities; the number of tree species was *X* = 4.8 before the activities and increased to *X* = 6.2 after the activities; and the total number of plants was *X* = 8.3 before the activities and increased to *X* = 17.6 after the activities. After participating in the activities, the number of herb and tree species recognized by the students increased. In addition, when the values obtained in the retest 4 months after the posttest are analyzed, it can be seen that the post‐test values increased slightly, with the average number of herbs being *X* = 11.8, the average number of trees being *X* = 6.5, and the total number of plants being *X* = 18.3. In this regard, it can be observed that students' knowledge of plant species continued to grow slightly.

**TABLE 2:** Average numbers of herb, tree, and total plant species identified by students.

	Herb	Tree	Total
Pretest	3.5	4.8	8.3
Posttest	11.4	6.2	17.6
Retest	11.8	6.5	18.3

The Pearson correlation coefficient was computed to assess the linear relationship between posttest QBB score and total average number of plant species identified by students. The two results were found to be strongly positively correlated, *r* (42) = .746, *p* = .000.

The students were asked where they had learned the names of Herb and Tree species following the activities. Students are permitted to submit from multiple sources. Therefore, the percentage values in the table indicate what proportion of the total of 44 students cited the title as a source.

As seen in Table 3, after completing the activities, the students cited the PlantNet mobile application as the source from which they had most learned about herb (77.3%) and tree (81.8%) species. In addition, the family (52.3% for herb and 54.5% for tree) and teachers (25% for herb and tree) were also important sources.

**TABLE 3:** Students' sources for learning herb and tree species.

	Herb		Tree	
	Frequency	%	Frequency	%
PlantNet	34	77.3	36	81.8
Family	23	52.3	24	54.5
Teacher	11	25	12	25
Books	1	2.3	2	4.5
Friends	0	0	1	2.3

## 4 DISCUSSION AND CONCLUSION

The biodiversity conservation and interest behaviors of the 5th‐grade secondary school students who took part in the PlantNet mobile application activities demonstrated a significant increase after the activities. Because they valued biodiversity, it is believed that the students were willing to transform their positive perspectives into behaviors. Similarly, there was a positive shift in students' attitudes toward biodiversity in studies where biodiversity education was provided through plant identification applications (Dolenc‐Orbanić et al., 2016; Echeverria et al., 2021; Gass et al., 2021; Iskrenovic‐Momcilovic, 2020; Unger et al., 2021). The students' increased knowledge about the variety of plants and plant species in their environment may be the cause of this improvement in the students' biodiversity conservation and interest behaviors. This conclusion is supported by the strong positive correlation between students' biodiversity conservation and interest behaviors and the number of plants they were able to learn. Previous research has found that as students' knowledge of the diversity of species increased, so did their interest in the environment and environmental issues (Erökten, 2006; Kaasinen, 2019).

It was noted that after the activities, a significant increase was seen in the number of herbs and trees the students could identify, which was initially quite low. A possible explanation for this is that city‐dwelling students, such as the participants in this study, have limited knowledge about plant species unless they receive education about biodiversity, and that the plant species they know about are typically food plants. In studies conducted in Switzerland with 8–16 year‐ old students (Lindemann‐Matthies, 2002), Germany with 13.8 average age (Lückmann & Menzel, 2014), and Finland with students from all age groups (Kaasinen, 2019), it was discovered that the number of plant species identified by the students participating was quite limited. In studies in which students were asked to list the plant species they knew, it was observed that students mostly mentioned the names of plants grown and cultivated at home, while they had very little knowledge about exotic and wild plant species (Huxham et al., 2006; Patrick & Tunnicliffe, 2011). The names of species of berries and trees were also well remembered (Kaasinen, 2019). In a different study conducted in France, it was found that students attached greater importance to the conservation of exotic species than to the conservation of local species, primarily because they were more familiar with exotic species due to media exposure (Ballouard et al., 2011). The students in this study cited the PlantNet mobile application as the most useful resource for learning about plant species following the activities. This result indicates that the activities served their intended purpose and that the students were better able to recognize the plant species in their environment as a result of using the mobile application. According to the students, they also learned about herb and tree species from their teachers and families. It is believed, however, that this learning was a result of students' increased awareness and curiosity following their use of the mobile plant identification application and that they had no interest in learning about plant species from these sources prior to this. The communication that students establish with their families as a result of the activities carried out with the mobile application may have other positive consequences. Talking about nature with their parents or friends positively influences students' willingness to conserve biodiversity and the frequency of their experiences in nature (Soga et al., 2016). In this regard, it can be said that mobile application activities led the students to seek out more information about plant species from various sources and increased the students' interest in biodiversity.

According to the QPS‐collected pre‐test data, students wrote the common names of plants but did not know their Latin names. In addition, in the 5th‐grade science curriculum (Ministry of National Education, 2018), it is recommended that students should not be taught the names of living things with systematic names. For this reason, the students wrote the names of the plants in their notebooks with their common names. The same preference was repeated in the posttest and retest data collected with QPS. Since the existing knowledge of the participant students about plants was in the form of common names, the students had the opportunity to improve their existing knowledge thanks to the applications made with the mobile plant identification application. In addition, it is thought that this naming method is more appropriate for the cognitive level of the students. However, if the same procedure is applied to students in higher grades, it would be more appropriate to record the plant names in Latin.

Although it was concluded that the activities had the desired effect on the students, the teacher who implemented the activities made some suggestions to improve them. At first, the instructor expressed a desire to allocate more time to the activities. Due to time constraints imposed by the science curriculum, the activities were conducted in a simplified manner and focused solely on plant names. In addition, the teacher stated that in plant identification activities, it would be more effective if the student could compare the results from the mobile application with those from another printed or electronic source, but this could not be done. Students need training in this subject to be able to identify plants through such materials, and this subject is not appropriate for their age group. Additionally, this is impossible due to time constraints. The teacher informed the class that they could also conduct research on the properties of the plants they had identified during the activities. However, it was observed that the students only recorded the names of the species and did not investigate their properties. Therefore, the teacher explained that in future activities, it would be more effective to provide students with resources to research plant characteristics and to ask them to conduct this research so that students could more effectively address the issue of plant blindness.

In addition, as a result of the activities, a positive and high‐level relationship was found between the students' behaviors, demonstrating an understanding of biodiversity, and the total number of plant species they knew. Identifying and learning about animal and plant species is an essential part of biodiversity education and a key indicator of knowledge about biodiversity (Lindemann‐Matthies, 2002; Palmberg et al., 2017; Randler, 2008; Wolff & Skarstein, 2020). Knowledge of different species extends beyond terminology; it is also about being able to identify species in their habitats, knowing how and where they exist, and understanding how they interact with other species (Skarstein & Skarstein, 2020). Therefore, it is expected that students who have knowledge about plant species will transform this knowledge into behaviors demonstrating an understanding of biodiversity and score higher on the QBB in this regard.

The involvement of individuals who are not professional scientists, such as students and ordinary citizens, in scientific research and data collection is currently referred to as citizen science (CS) (Holmgren, 2020). Currently, CS projects often rely on ICT as they enable easy access to participants and streamline data collection (Preece, 2016). A paradigm shift is occurring in the future of biodiversity research due to CS (Callaghan et al., 2021). PlantNet exemplifies a range of projects that concentrate on biodiversity and utilize ICT. Given that participants in CS projects do not receive any form of compensation, the issue of motivating them to gather data becomes a significant challenge. Gamification is regarded as a potent strategy for enhancing participant motivation and data collection in CS projects (Bowser et al., 2014; Holmgren, 2020). A study on Biotracker, a gamified mobile application used to collect data about plants, revealed that users who lack intrinsic motivation to perform the activity of verifying and observing plant data are motivated by gamified elements (Bowser et al., 2014). Torres‐Toukoumidis et al. (2022) examined 10 ecology‐oriented mobile applications in terms of gamification elements categorized under the headings “Components, Mechanics and Dynamics,” but PlantNet was not among the mobile applications examined. Nevertheless, the PlantNet mobile application can be considered to possess gamification elements to some extent, based on these criteria. In this respect, the motivation of students to use the application and the extent to which this motivation is caused by the gamification features of PlantNet should be investigated in future studies.

As a result of the study, it was determined that the activities conducted with the mobile plant identification application during out‐of‐school learning were an effective technique that can be used in biodiversity education. Therefore, it is recommended that promotional activities be conducted to increase the method's popularity among educators. The method can be promoted on social media and introduced during in‐service training activities for teachers. In addition, teachers and prospective teachers can be informed about this method, and it can be incorporated into innovative teaching approaches. In the future, it is recommended that experimental studies be conducted to add to the data on the use of this method in biodiversity education. Moreover, in experimental studies, mobile technology‐based biodiversity activities could be further expanded into project‐based studies.

## AUTHOR CONTRIBUTIONS

**Ozan Coşkunserçe:** Conceptualization (lead); data curation (lead); formal analysis (lead); investigation (lead); methodology (lead); resources (lead); software (lead); supervision (lead); validation (lead); visualization (lead); writing – original draft (lead); writing – review and editing (lead).

## FUNDING INFORMATION

None.

## CONFLICT OF INTEREST STATEMENT

There is no conflict of interest to declare.

## Supporting information


Data S1

## Data Availability

The data that support the findings of this study are available from the corresponding author upon reasonable request. Coşkunserçe, O.; 2023; Use of a Mobile Plant Identification Application and the Out‐Of‐School Learning Method in Biodiversity Education; Apertera; Sürüm 1; https://aperta.ulakbim.gov.tr/record/252527.
